# Geographical Origin, WASH Access, and Clinical Descriptions for Patients Admitted to a Cholera Treatment Center in Northwest Syria between October and December 2022

**DOI:** 10.1055/s-0043-1776045

**Published:** 2023-10-30

**Authors:** Ahmad Hmaideh, Maia C. Tarnas, Wasim Zakaria, Ahmad Oussama Rifai, Mosab Ibrahem, Yaser Hashoom, Nusaima Ghazal, Aula Abbara

**Affiliations:** 1 Syrian Board of Medical Specialties, Syria; 2 Syria Public Health Network, United Kingdom; 3 Department of Population Health and Disease Prevention, University of California Irvine, Irvine, California, United States; 4 The Virtual Nephrologist, Florida, United States; 5 Department of Infectious Diseases, Imperial College, London, United Kingdom

**Keywords:** cholera, conflict, Syria, outbreaks, WASH, water and sanitation

## Abstract

**Background** On September 10, 2022, a cholera outbreak was declared in Syria for the first time in over a decade of protracted conflict. As of May 20, 2023, 132,782 suspected cases had been reported, primarily in northwest and northeast Syria. We aim to provide a detailed description of water sources and clinical status of a patient cohort seen at a cholera treatment center (CTC) in northwest Syria.

**Methods** We retrospectively identified patients with confirmed cholera who presented to the CTC in Idlib governorate between October 8 and December 18, 2022. Data were obtained from clinical case records and analyzed in R v4.0.4.

**Results** Ninety-four patients (55.3% men) were treated at the CTC. Thirty-five patients were severely dehydrated (Plan C treatment), 54 had some dehydration (Plan B), and 5 had no dehydration (Plan A). Most patients were between 11 and 20 years old ( *n* = 25, 26.6%) or 31 and 40 years old ( *n* = 19, 20.2%). Note that 70.2% ( *n* = 66) of patients were seen in November 2022 and most were from Harim district ( *n* = 44, 46.8%). Public wells ( *n* = 46, 48.9%) and water trucking ( *n* = 41, 43.6%) were the most commonly used water sources. Note that 76.6% ( *n* = 72) did not have access to chlorine-treated water. Forty-seven patients (50%) had more than five water, sanitation, and hygiene (WASH)-related cholera risk factors. Following treatment, six patients were transferred to another treatment center, three died (case fatality rate: 3.2%), and the remainder were discharged.

**Conclusion** Most patients reported WASH-related risk factors for cholera, reflecting the poor state of WASH in northwest Syria after over a decade of conflict. This relates to the direct and indirect impacts of urban and periurban violence as well as the underfunded humanitarian response. Strengthening WASH and health promotion are important components to control the outbreak.

## Introduction

For many years, there has been concern in Syria that an outbreak of cholera could occur given the poor state of water, sanitation, and hygiene (WASH) across the country from the protracted conflict, climate change-related droughts and floods, and insufficient water quality and quantity to meet the population's needs. 1 The conflict has caused public health, which was weak even before the conflict, to further deteriorate. 2 In August 2022, there was an increase in cases of acute watery diarrhea (AWD), the syndrome associated with cholera, in Aleppo governorate and in the northeast of Syria. As of May 20, 2023, there have been 132,782 suspected cases with Idlib (35%) and Aleppo (29%) in northwest Syria and Deir Ez-Zor (16%) and Ar-Raqqa (16%) in northeast the most affected. 3

Syria's conflict has led to the emergence of multiple subnational health systems; these have evolved differently across the country due to varying population needs, governance structures, and resources. 2 In northwest Syria, humanitarian organizations (both local and international) have provided important support to primary and secondary care services. This area has seen among the most intense conflict with deliberate targeting of hospitals. 4 Additionally, there has been targeting and interruption of WASH; the summer of 2020 saw the greatest interference of WASH in Idlib, with multiple attacks by the Syrian regime and its allies interrupting water to vulnerable populations in the area. 5

In February 2023, cholera control in the area was further affected by the severe earthquakes in southeastern Türkiye and northern Syria that caused increased forced displacement, over 8,000 deaths, and interrupted cholera control measures. 6 However, in early March 2023, an oral cholera vaccine campaign that had been delayed due to the earthquakes resumed and ultimately reached 1.7 million people in the area. 7

Our aim is to provide a detailed description of water sources and clinical status of a cohort of patients seen at a cholera treatment center (CTC) in northwest Syria during the 2022 outbreak.

## Materials and Methods

### Study Area and Setting

Northwest Syria comprises Idlib and parts of Aleppo governorates, which are outside of Syrian government control. Their estimated population is around 4.2 to 4.6 million individuals, of whom more than 65% are internally displaced. 8 Around 1.5 million live in tented settlements with poor access to WASH. This area has experienced severe bombardments by the Syrian government and its allies including to hospitals and WASH infrastructure. 5 This has further worsened access to water and health care, which is similarly compounded by widespread poverty. The targeting of health and civil infrastructure has contributed to the reemergence and spread of diseases such as cholera, tuberculosis, and leishmaniasis that were rare before the conflict. The fragile health system in northern Syria is insufficient to meet the growing needs of the population: there are too few hospitals for a large number of people and insufficient laboratory equipment to aid in the diagnosis of diseases. For example, there is only one laboratory that can culture stool in Idlib governorate.

Following the appearance of AWD cases in Idlib, a CTC was established in Darkoush following the World Health Organization guidelines. The CTC had 2 wards with 10 beds each, and an isolation chamber in which patients were initially examined. In addition to doctors and nurses, the CTC included a health awareness team that taught people about cholera, how it is transferred, and how to prevent its spread. Following standard practice, patients with severe dehydration and danger signs received Plan C treatment, patients with some dehydration but no danger signs received Plan B treatment, and patients with no dehydration received Plan A treatment. 9

### Data Collection

Patient information was originally collected by the CTC medical staff during routine patient intake following admission to the CTC and a positive stool culture sample from October 8, 2022 to December 18, 2022. Data included information on demographic variables, the clinical case, and treatment. These data were deidentified prior to the research team obtaining them. Cholera case severity was classified according to the Global Cholera Task Force staging where Plan A is no signs of dehydration, Plan B is some signs of dehydration, and Plan C is severe dehydration. 9

### Analysis

In this report, we included all patients ( *n* = 94) admitted to the CTC. Patient ages were aggregated into age groups of < 2 years old, 2 to 10 years old, and groups of 10 years up to age 80. Analysis was done in both Arabic and English using R v.4.0.4.

### Ethics Statement

This study was deemed exempt by the Human Research Protections board at the University of California, Irvine.

## Results

**Fig. 1:**
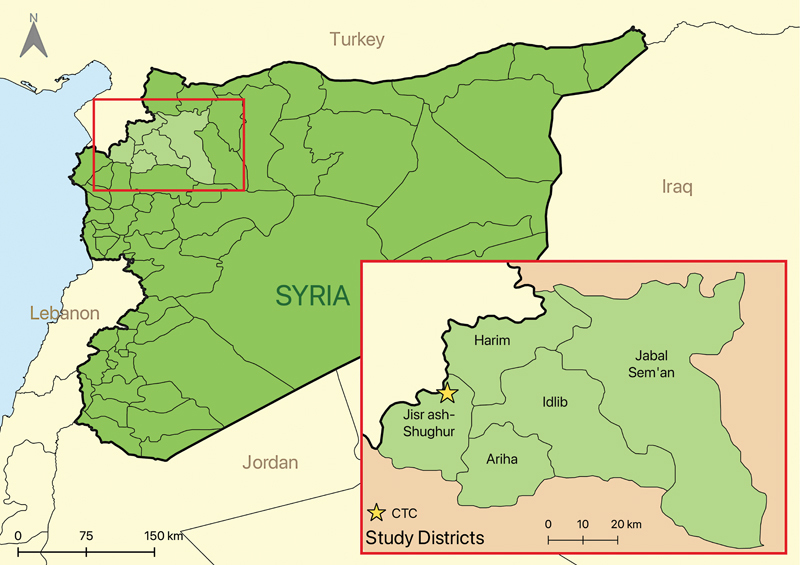
Map of Syria with cholera treatment center (CTC) patients' districts highlighted. Note that Atma camp is located in Harim district. Figure made with QGIS 2.33.1.

Patient characteristics can be found in Table 1 . The majority of patients (70.2%; 66/94) were admitted to the CTC in November 2022 and were from the Harim district of Idlib (47%; 44/94), followed by Idlib district (19%; 18/94), Jisr ash-Shughur district (16%; 15/94), and Atma camp (15%; 14/94) ( Fig. 1 ).

**Table 1:** Demographic details, risk factors for cholera, and patient treatment classification for the 94 patients included in this study

Patient characteristics	Female ( *n* = 42)		Male ( *n* = 52)		Total ( *n* = 94)	
Demographics	*n*	%	*n*	%	*n*	%
Age, y						
< 2	3	(7)	3	(6)	6	(6)
2–9	9	(21)	16	(31)	25	(27)
10–19	4	(10)	10	(19)	14	(15)
20–29	5	(12)	3	(6)	8	(9)
30–39	10	(24)	9	(17)	19	(20)
40–49	3	(7)	3	(6)	6	(6)
50–59	6	(14)	3	(6)	9	(10)
60–69	2	(5)	1	(2)	3	(3)
70–80	0	(0)	4	(8)	4	(4)
Living type						
Village	17	(40)	15	(29)	32	(34)
City	16	(38)	19	(37)	35	(37)
Camp	9	(21)	18	(35)	27	(29)
Risk factors *						
Not washing food	42	(100)	51	(98)	93	(99)
Poor sanitation	32	(76)	35	(67)	67	(71)
No sewage network	29	(69)	35	(67)	64	(68)
No clean water	27	(64)	35	(67)	62	(66)
Contact with cholera patient	26	(62)	28	(54)	54	(57)
Travel to endemic area	23	(55)	25	(48)	48	(51)
Ate uncooked fish	0	(0)	1	(2)	1	(1)
Patient treatment						
Plan A	0	(0)	5	(10)	5	(5)
Plan B	24	(57)	30	(58)	54	(57)
Plan C	18	(43)	17	(33)	35	(37)

Note: Patients with severe dehydration and danger signs received Plan C treatment, patients with some dehydration but no danger signs received Plan B treatment, and patients with no dehydration received Plan A treatment.

* Patients could face multiple risk factors.

Seventy-eight percent (73/94) of patients had less than a secondary school education, with 41% (39/94) considered illiterate. Forty-five (48%) were 19 years old or under and 21 (22%) were students. Thirty (61%) were homemakers and 8 (17%) were government workers.

Across all patients, water from public wells was most frequently used for all water purposes, but water sources varied across residential locations ( Table 2 ). Patients from camp settings relied primarily on trucked water, whereas patients from cities used public wells and patients from villages often used both public wells and water trucks. Seventy-two (77%) patients did not use chlorine to treat water with 70 stating they did not have access to chlorine. Only two patients treated water by boiling.

**Table 2:** Water sources for drinking, food preparation, bathing, and washing by living situation for the 94 cholera patients included in this study

	Drinking			Food preparation			Bathing			Washing		
	Camp ( *n* = 27)	City ( *n* = 35)	Village ( *n* = 32)	Camp	City	Village	Camp	City	Village	Camp	City	Village
Source												
Public well												
* n*	1	30	14	1	30	14	2	31	14	3	30	15
%	(4)	(86)	(44)	(4)	(86)	(44)	(7)	(89)	(44)	(11)	(86)	(47)
Water truck												
*n*	25	2	15	24	2	15	24	2	15	24	2	14
%	(93)	(6)	(47)	(89)	(6)	(47)	(89)	(6)	(47)	(89)	(6)	(44)
Borehole												
*n*	1	3	3	2	3	3	1	2	3	0	3	3
%	(4)	(9)	(9)	(7)	(9)	(9)	(4)	(6)	(9)	(0)	(9)	(9)

### Clinical Characteristics

Upon admittance to the CTC, the median patient heart rate was 110 (interquartile range [IQR]: 30), oxygen saturation level was 97% (IQR: 3), systolic blood pressure was 100 (IQR: 10), and diastolic blood pressure was 70 (IQR: 0). All patients but one were given a rapid cholera test, on which all but two patients tested positive. All patients had diarrhea, 93 (99%) were vomiting, 88 (94%) experienced abdominal pain, and 2 (2%) had a fever. Most patients (52%; 49/94) were experiencing diarrhea 5 to 10 times a day or more than 10 times a day (44%; 41/94). Few patients reported comorbidities: 5 reported diabetes, 5 reported hypertension, 3 reported heart failure, and 1 patient was pregnant.

Patients were treated using oral rehydration salts (94%; 88/94), intravenous fluids (91%; 86/94), oral or intravenous zinc sulfate (84%; 79/94), and oral or intravenous antibiotics (70%; 66/94). Most patients (66%; 62/94) received all four treatments. Antibiotic choices were based on Early Warning and Response Network (EWARN) microbiological confirmation of sensitivity. For antibiotics, azithromycin was used most frequently (62%; 41/66), followed by ciprofloxacin (32%; 21/66).

### Outcomes

Patients remained in the CTC for an average of 2.7 days (range: 1–7 days). Of the 94 total patients, 6 were transferred to another treatment center and 3 died (case fatality rate: 3.2%). Of the 6 who were transferred, 1 was because of gastrointestinal bleeding, 1 because of acute electrolyte imbalance, 1 because of appendicitis, and 3 patients did not have a reason listed. Of the 3 patients who died, 2 received Plan B treatment (some dehydration) and 1 received Plan C treatment (severe dehydration). The causes of death differed: one died of metabolic acidosis and electrolytes disorder, another from multiple organ dysfunction syndrome, and the third cause of death is unknown following transfer to another hospital. The remainder were discharged.

## Discussion

This study highlights the poor availability of WASH for patients presenting to the CTC with confirmed cholera as well as the geographical variation across Idlib governorate. Harim district accounted for 47% of cases though proximity to the CTC (25 km) may have led to Harim residents being overrepresented. However, when overall cases in northwest Syria are noted (86,404 as of May 29, 2023), Harim accounts for the largest number of cases and 29.5% of all recorded cases in the area. 10 Importantly, following a surge in patients from Harim at the CTC, the CTC alerted local health officials of the cases and prompted an investigation. They were told of a leak from the sewage network which contaminated the water sources used for drinking and a reduction in available chlorine.

Our study also shows a high prevalence of risk factors, in particular of not washing food (which 99% of cases reported); this is likely compounded by other relevant factors such as poor availability of clean water (reported by 66%) and contact with a cholera patient (57%). Though Risk Communication and Community Engagement have been key activities in response to the cholera situation in northwest Syria, this suggests that more can be done to provide the local population with the means to protect themselves and their families. The absence of adequate WASH in northwest Syria has been highlighted at both the macro 5 and micro 11 levels; the latter has been particularly relevant during the coronavirus disease 2019 pandemic.

Though there was a slightly higher proportion of men in this cohort (55%), it is notable that a larger proportion of females were in the Plan C treatment group. This warrants further exploration, particularly as males and females in Plan B are similar at 57%. In northwest Syria broadly, females are 48% of cases, though more detail about the severity is not available. 10 Though cholera is described as an “equal opportunity” infection, there is important literature on the gendered aspects of cholera susceptibility and control. Reports from Sierra Leone discuss how gender roles and differing social responsibilities may lead to differential interactions with cholera. 12 A 2010 United Nations International Children's Emergency Fund briefing note on gender and the cholera outbreak in Haiti also noted the importance of integrating gender considerations in cholera response. 13 They reference prior studies in Indonesia and Kenya which noted higher rates of cholera in adult females and school-aged girls as well as higher case fatality and morbidity among females in studies from South Africa and Bangladesh. 14 15 16 17 18 19 Owing to social and gender norms, females play important roles in both prevention and response. For example, a key prevention method is the boiling or treatment of water which, for the most part, falls to women and girls. Consideration must therefore be given to the gender lens as it relates to the sociocultural, economic, and environmental factors that may contribute to an individual's risk of disease.

Despite public health campaigns including the distribution of soap and chlorine, most of the 77% of patients who reported not using chlorine to treat water reported that they did not have access to chlorine. It is unclear whether this was due to intermittent distributions, a lack of sustainable distributions, and/or if there were incomplete distributions. An alternative to chlorine is boiling water, which only two patients reported doing; it is likely that this is not a sustainable intervention in the overcrowded and underresourced settings of northwest Syria, especially with limited fuel resources. These findings highlight the importance of sustained provision of chlorine tablets or other equivalent water purifiers, especially given the unreliability of water cleanliness. Such tablets are often included in humanitarian outbreak response, 20 but proactive and uninterrupted provision would be more useful for preventing future outbreaks.

The sources of water for drinking, food preparation, bathing, and washing show expected variability between camp, city, and village settings. Camp settings rely heavily on water trucking for all usages compared to the others. This is unsurprising, given that camps frequently lack sufficient WASH infrastructure, and especially infrastructure that can withstand harsh camp conditions. Private water trucking is not heavily regulated 21 and can cost a high proportion of a household's daily income. Well water was the most used in the cities, and villages used a roughly even split between public wells and water trucking. Importantly, access to a public well does not infer that the water at the well is suitable for drinking, 22 as evidenced by this outbreak. Because of the price of trucked water, however, many individuals do not have an alternative.

The association between conflict, insecurity, and cholera has been described previously in the literature with examples from Yemen, 23 Nigeria, 24 and Haiti. 25 In Syria, not only has the conflict adversely affected WASH infrastructure, 5 but associated poverty 26 has also affected the population's ability to ensure safe access to water, particularly for children. 27

We present findings from a single CTC and, as such, the data may not represent the situation or patient demographics found in other CTCs. The presence of other CTCs in northwest Syria may also affect the representation of reported age ranges and living situations. Our clinical data for some patients is also incomplete, which prevents us from providing full case details for each patient. This is particularly of note for the two Plan B patients who died (as this would be unexpected if the patients did not first become Plan C) and the patients who were transferred to another health facility. However, we are able to provide complete clinical details for most patients' cholera cases. There may be risk of social desirability or recall bias in several of the risk factor and water procurement questions. However, because of the high rates of positive responses for certain risk factors, we do not believe that this bias was prominent.

This piece offers important insight to the ongoing cholera outbreak in northern Syria. We are able to report on the demographic makeup of patients, their clinical outcomes, water sources, and relevant risk factors. In doing so, we can illustrate the differences, especially in water sources, between patients residing in distinct areas (camp, city, and village) and between sexes. This can be especially useful in outbreak response, as a one-size-fits-all approach may not be appropriate in such settings. Indeed, without being receptive to differences across water sources and risk factors, outbreak response may miss key transmission routes or risk factors in specific groups. The insights presented in this study also emphasize the need for routine provision of chlorination tablets or other water purifiers, especially given that boiling water is often infeasible for many. Cholera is entirely preventable, though Syria's protracted conflict has resulted in conditions that are extremely hospitable to infectious diseases. Depoliticization of WASH, health care, and humanitarian aid, in addition to more robust prevention measures, are necessary to prevent future outbreaks of this sort.
